# Selective activation of nerve fiber subpopulations with intrafascicular stimulation

**DOI:** 10.1088/1741-2552/aea369

**Published:** 2026-09-24

**Authors:** Arianna Ortega Sanabria, Louis Regnacq, Anil K Thota, Avery Holmes, Justin M Asbee, Sylvie Renauld, Florian Klobl, Yannick Bornat, Samantha Robinson, Laura McPherson, James J Abbas, Ranu Jung

**Affiliations:** 1 Institute for Integrative and Innovative Research, University of Arkansas, Fayetteville, AR, United States of America; 2 Department of Biomedical Engineering, University of Arkansas, Fayetteville, AR, United States of America; 3 IMS Lab, UMR 5218, University Bordeaux, Bordeaux INP, Talence, France; 4 Center for Agricultural Data Analytics & School of Human Environmental Sciences, University of Arkansas, Fayetteville, AR, United States of America; 5 Program in Physical Therapy and Department of Neurology, Washington University School of Medicine in St Louis, Saint Louis, MO, United States of America

**Keywords:** selective nerve stimulation, longitudinal intrafascicular electrodes, peripheral nerve stimulation, intrafascicular selectivity, bioelectronics

## Abstract

*Objective.* Peripheral nerve stimulation (PNS) is most effective when specific nerve fiber subpopulations are activated, while minimizing off-target activation, which may cause undesirable side effects. This selectivity depends primarily on electrode design and charge delivery, but it remains unclear how selectivity depends on electrode location within a fascicle and whether intrafascicular field steering can shape recruitment of different nerve fibers. Our objective was to test the hypothesis that selective PNS could be achieved through electrode placement and intrafascicular electric field steering using longitudinal intrafascicular electrodes (LIFEs). *Approach.* LIFEs were implanted into the tibial fascicle of the sciatic nerve of 17 anesthetized adult rats. We tested whether electrodes positioned at different cross-sectional and longitudinal locations within the same fascicle, together with different electric field-steering approaches produced distinct activation patterns in the gastrocnemius lateralis muscle. Muscle responses were measured using high-density epimysial electromyography (HD-eEMG). *Main Results.* Electrodes placed at different locations within the same fascicle activated distinct muscle regions, demonstrating intrafascicular selectivity. Bipolar stimulation recruited nerve fibers differently than monopolar stimulation, showing that electric field steering can further shape the selective recruitment. In both configurations, increasing the stimulation amplitude produced a graded increase in muscle activation. Furthermore, our findings demonstrated that HD-eEMG is an effective tool for evaluating intrafascicular selectivity. *Significance.* These findings suggest that improving on-target selectivity may support next-generation bioelectronic therapies with better outcomes and fewer side effects, potentially enabling more precise, organ-specific neuromodulation. Using multiple intrafascicular electrodes may provide two complementary strategies for enhancing selectivity: strategic intrafascicular placement to access different fiber subpopulations and bipolar configurations to steer recruitment beyond what a single electrode can achieve.

## 1. Introduction

Peripheral nerve stimulation (PNS) is a promising therapy for conditions inadequately managed by pharmaceuticals or physical rehabilitation, including depression, epilepsy, lower back pain, and bladder or fecal incontinence [1–4]. However, its clinical utility is limited by its off-target effects. For example, vagus nerve stimulation (VNS) can treat depression [1, 5] and reduce epileptic episodes [3]; however, side effects, such as hoarseness and neck pain, often lead to discontinuation [4]. Similarly, sacral nerve stimulation in inflammatory bowel disease and urinary or fecal incontinence [6] can cause abnormal sensations, abdominal pain, altered intestinal function, and pelvic tingling [2]. In individuals with upper-limb amputations, cuff-based PNS can evoke useful sensory percepts but may also trigger unwanted motor activity [7].

A major cause of these off-target effects is limited stimulation selectivity, which leads to the unintended activation of non-targeted nerve fibers. This limitation is partly due to an incomplete understanding of nerve structure and organization [8–10]. Selectivity is defined as the ability to activate one population of neurons without activating neighboring, but unwanted, populations [8]. Improving selectivity is therefore essential to reduce side effects, improve outcomes, and enable more precise, organ-specific neuromodulation therapies [8, 11, 12].

Neurostimulation selectivity can be categorized into two primary types: fiber-type and topological selectivity. Fiber-type selectivity refers to the preferential activation of specific classes of nerve fibers, such as motor or sensory fibers. In contrast, topological selectivity, which is the ability to activate nerve fibers located close to the stimulating electrode irrespective of the fiber type, involves spatially targeted stimulation, either at the level of fascicles (interfascicular selectivity) or within subpopulations of fibers in a single fascicle (intrafascicular selectivity) [8, 13]. This study focuses on intrafascicular topological selectivity.

Selectivity also depends on the neural interface, including intraneural, extraneural, or surface electrodes [8, 14, 15]. Although extraneural cuff electrodes are widely used because they are easy to implant and are clinically effective, their distance from the target fibers limits their selectivity. In contrast, positioning electrodes closer to the target population improves selectivity [8, 11, 16]. Consequently, intraneural electrodes, such as longitudinal intrafascicular electrodes (LIFEs), are particularly promising for enhancing intrafascicular selectivity because they are placed within the fascicle near specific fiber groups [11, 17, 18]. Although previous studies have shown that LIFEs can provide greater selectivity than extraneural electrodes [19], several key questions remain unresolved. Specifically, responses elicited by individual electrodes within the same fascicle have not been directly compared, and the effects of stimulation amplitude on selectivity have not been characterized.

Another approach to improving interfascicular selectivity is extraneural electrical field steering, which uses one or more adjacent electrodes arranged in multipolar configurations with simultaneous independently controlled stimulation at each pole to shape the electrical field more precisely [20, 21]. This approach can selectively activate different fascicles and improve interfascicular selectivity [21–24]. However, targeting centrally located fascicles remains limited and has not been shown to substantially improve intrafascicular selectivity [25]. Whether field steering using multiple LIFEs can achieve selective activation of nerve fibers within a single fascicle has not been directly tested.

In this study, we investigated the effects of electrode placement and intrafascicular field steering on nerve fiber recruitment using single and multiple LIFEs implanted within the tibial fascicle of the sciatic nerve, directly addressing whether individual electrodes within the same fascicle elicit different patterns of activation and whether bipolar stimulation can access different sets of fibers within a fascicle. High-density epimysial electromyography (HD-eEMG) arrays were used to assess the spatial distribution of resulting muscle activation patterns, which can indicate changes in the set of motor fibers activated. Our results demonstrate that deploying multiple LIFEs within the same fascicle in a monopolar configuration enables selective activation of distinct subpopulations of nerve fibers, resulting in different spatial muscle activation patterns that display the distribution of evoked responses from the muscle fibers under the HD-eEMG grid array. Focused activation reflects evoked muscle activity that is concentrated in fewer HD-eEMG channels across the grid. Furthermore, using multiple LIFEs in a bipolar configuration enables field steering, shifting the focus of activation, and altering the spatial muscle activation pattern.

These findings indicate that electrode placement within the fascicle and field steering can enhance intrafascicular selectivity. This approach has the potential to advance focal neural stimulation and support the development of organ-specific neuromodulation therapies, with minimal or no side effects.

## 2. Methods

We investigated two strategies for enhancing intrafascicular stimulation selectivity using LIFEs. Specifically, we assessed the effect of positioning multiple LIFEs within the same fascicle and using multiple LIFEs within the same fascicle arranged in a bipolar configuration to steer the electric field. The experiments were performed on 17 adult male Sprague–Dawley rats (433 ± 29 g) under anesthesia. All animal care and handling procedures were conducted in accordance with protocols approved by the Institutional Animal Care and Use Committee of the University of Arkansas (Protocol AUP22014).

### 2.1. Electrode fabrication

Custom LIFEs were fabricated from insulated Pt/Ir wires (27.5 *µ*m in diameter) with an active site of approximately 1 mm created by de-insulating the wire. One end of the wire was welded to a tungsten needle (75 *µ*m in diameter) to facilitate fascicular implantation, whereas the opposite end was welded to a metal plate to connect to the stimulator. The electrode impedance was verified using a custom bioimpedance measurement system [26], ensuring values within 8–40 kΩ (mean: 16.9 ± 9.5 kΩ) at 1 kHz. From these recordings, we estimated the active site length of the electrodes to be 550 ± 100 *µ*m [27].

### 2.2. Surgical procedures and electrode implantation

Anesthesia was induced and maintained with inhaled isoflurane (1%–3% ISO in O_2_, flow rate approximately 3 l min^−1^). The sciatic nerve was exposed via a lateral thigh incision, and the surrounding muscles were gently retracted using elastic stays to isolate the nerve.

**Figure 1.**
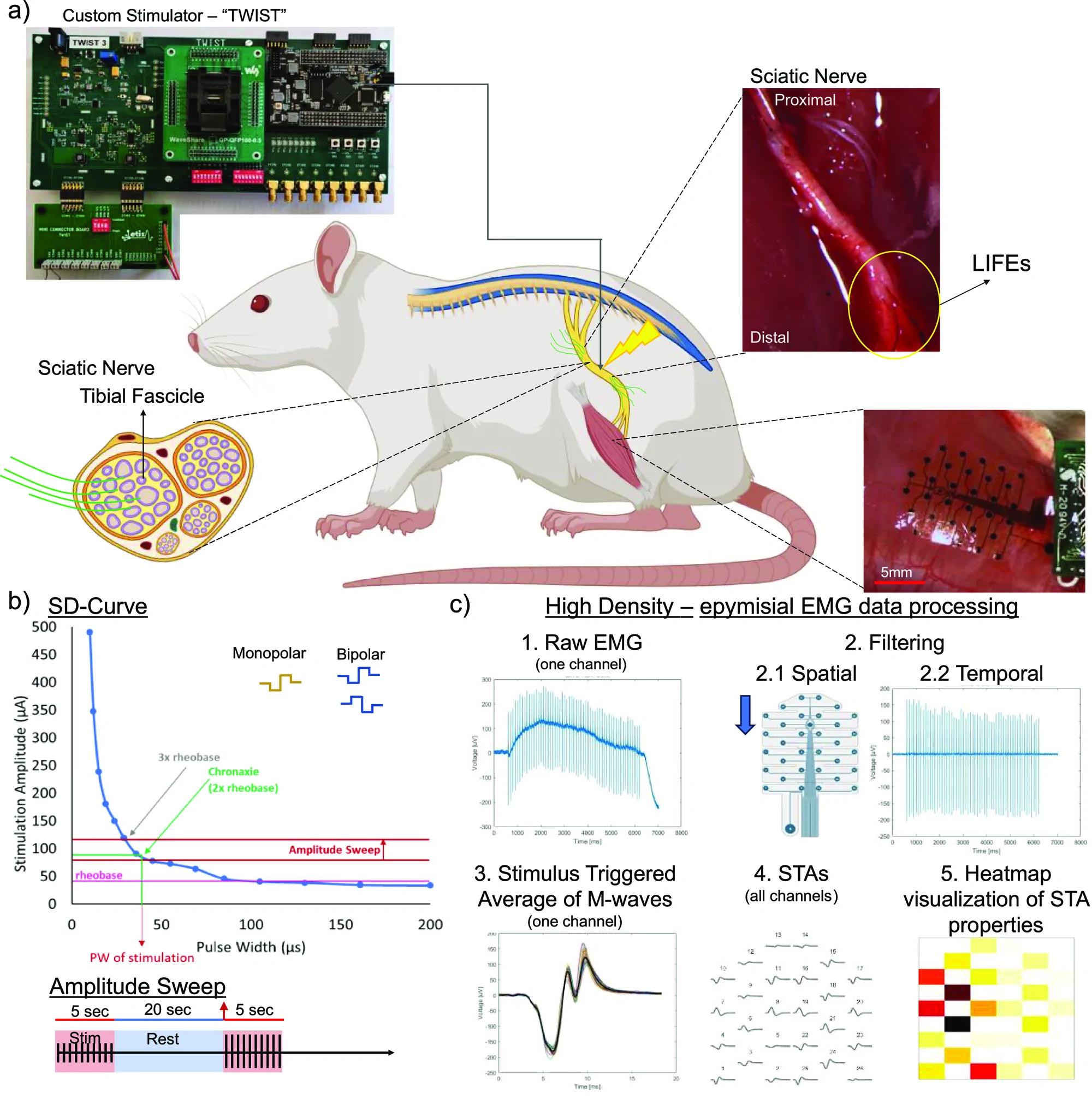
*Study design*. (a) Schematic: A custom stimulator ‘TWIST’ generated electrical stimulation pulses delivered to the tibial fascicle of the sciatic nerve using longitudinal intrafascicular electrodes (LIFEs). High-density epimysial electromyograms (HD-eEMG) are collected from the gastrocnemius lateralis muscle with a flexible array. Schematic partially made with Biorender.com (b) Strength-duration (SD)-curves were used to identify the rheobase and chronaxie for each electrode and stimulation configuration. To generate SD-curves, pulses at 1 Hz were delivered at each pulse width and the stimulation amplitude was increased until a muscle twitch was visible. The amplitude sweep protocol consisted of stimulation pulses delivered in monopolar or bipolar configurations in bursts of 5 s interspersed with 20 s rest to prevent muscle fatigue, with increase in pulse amplitude in subsequent bursts. (c) The HD-eEMG raw signals were spatially and temporally filtered. Subsequently, stimulus-triggered averages (STA) were calculated from the M-wave responses for each HD-eEMG channel. The root mean square (RMS) amplitude derived from the STA was used to generate heatmaps, providing a visual representation of the spatial distribution of muscle activation.

Figure 1 illustrates the design of the *in vivo* experimental study. LIFEs were implanted into the tibial fascicle of the sciatic nerve (2–4 electrodes per rat; figure 1(a)), aligned parallel to the axons, by inserting the needle from the proximal to distal end using a custom implantation tool. Appropriate placement and electrode functionality were verified by eliciting paw movements using a custom-built stimulator [28] that delivered biphasic, symmetrical square pulses. Electrodes that failed to evoke paw movement were excluded from stimulation. Electrodes were secured to the epineurium using 8–0 non-absorbable sutures (DemeTECH®). The paw was stabilized using a 3D-printed fixture.

Body temperature was continuously monitored using a rectal thermometer, maintained throughout the experiment with a heating pad (Kent Scientific, Inc.), and supplemented with hand warmers as needed. Heart rate, respiratory rate, and SpO_2_ were continuously monitored (Vetcoder). To prevent dehydration, 2 ml of saline was administered subcutaneously every two hours. At the conclusion of each experiment, the animals were euthanized by isoflurane overdose, with exposure maintained for at least 1 min after cessation of respiration, in accordance with AVMA guidelines. The experimental sessions lasted 6–11 h, from anesthetic induction to euthanasia, with electrode implantation and recordings typically completed within 4–6 h.

### 2.3. High-density epimysial EMG recordings

The fascia of the hindlimb was excised to reveal the gastrocnemius muscles, allowing placement of a 32-channel flexible high-density electrode array grid (‘Rat EEG Triangular H32,’ NeuroNexus Inc.) on the surface of the gastrocnemius lateralis. The array was positioned distally to proximally and aligned with the muscle innervation (figure 1(a)). Monopolar EMG signals were acquired using a Nano2 headstage (Ripple Neuro, Inc.) and sampled at 30 kS s^−1^. All raw data were recorded and stored using a digital acquisition system (Scout, Ripple Neuro, Inc.) for subsequent offline analyses. Trigger signals were transmitted from the stimulator to the digital acquisition system concurrent with stimulation delivery, ensuring precise synchronization between stimulation events and HD-eEMG recordings, thereby supporting comprehensive post-processing and analysis.

### 2.4. Strength-duration (SD) curves and stimulation thresholds

The stimulation thresholds for each implanted electrode were determined from the SD curves. Stimulation consisted of biphasic, square, cathodic-first pulses delivered at 1 Hz, utilizing either monopolar or bipolar configurations (figure 1(b)). For each pulse width (PW) tested, ranging from 20 *µ*s to 300 *µ*s, the stimulation current (pulse amplitude, PA) was incrementally augmented until a visible muscle twitch was observed. The minimum current required to elicit a muscle twitch was defined as the activation threshold. This procedure was repeated across all electrodes for each stimulation strategy under evaluation. Electrodes that did not produce muscle twitches were excluded from further analysis.

### 2.5. Stimulation protocol—amplitude sweep

Rheobase was determined from the corresponding SD curves for each individual electrode or electrode combination. The stimulation PW was selected on the basis of the chronaxie value, as shown in figure 1(b). For each electrode or electrode combination, the baseline stimulation level (threshold) was defined as the pulse amplitude associated with the selected PW.

To assess the recruitment of muscle fibers via monopolar or bipolar stimulation, recruitment profiles were generated by incrementally adjusting the pulse amplitude in steps of 2 *µ*A or 4 *µ*A. This evaluation commenced at two increments below the activation threshold. Because the step size was fixed while the activation threshold varied across electrodes, the number of amplitude levels ranged from 7 to 66. For most electrodes (38 out of 49, 13 rats), the sweep extended to 1.5xTH. For a subset of electrodes (11 out of 49, 4 rats), the sweep extended to 2.1xTH, as illustrated in figure 1(b). Stimulation consisted of biphasic, charge-balanced, square pulses in a monopolar or bipolar configuration (schematic shown in figure 1(b)). For monopolar stimulation, cathodic-first pulses were applied through a single LIFE, while for bipolar stimulation, two LIFEs were used, with one delivering cathodic-first pulses and the other delivering anodic-first pulses simultaneously. Stimulation was applied at 10 or 15 Hz in 5 s trains, with each train followed by a rest period of 20–30 s to minimize muscle fatigue. High-density EMG signals were continuously recorded throughout the stimulation protocol.

### 2.6. Data processing

Monopolar EMG recordings were spatially filtered to decrease the detection volume and isolate the electrical activity of the underlying muscle, as described by Gallina *et al* [29] (figure 1(c)). A single-differential spatial filter was applied along the direction of the muscle fibers (i.e. the columns) by subtracting each channel from its immediately adjacent channel in the column direction (i.e. Channel _(*r,c*)_ = Channel _(*r,c*)_—Channel_(*r*+1,*c*)_, where *r* denotes the row and *c* denotes the column). The resulting 26 channels of spatially filtered data were then filtered in the frequency domain using a fourth-order Butterworth bandpass filter with cut-off frequencies of 10 and 1000 Hz. Data from two HD-eEMG channels in one rat were excluded from the analysis because their signals were dominated by recording noise and did not exhibit physiologically meaningful variation.

For each stimulation level, a stimulus-triggered average (STA) of eEMG was calculated for each HD-eEMG channel to construct M-wave responses. To characterize these responses, the root mean square (RMS) amplitude of each STA M-wave was computed across a 16 ms window, beginning with the onset of the evoked response.

To evaluate the spatial distribution of eEMG amplitude (rather than the magnitude) across the electrode grid, we normalized STA RMS values for all eEMG channels for a given electrode configuration and stimulation level to the highest of those RMS values. Spatial heatmaps of stimulation-evoked M-wave amplitude were constructed from the normalized RMS values for data visualization (figure 1(c)). Next, eEMG channels were sorted in descending order of their normalized RMS values, which were then plotted against the sorted channel numbers. This created an amplitude rank profile for each stimulation level and electrode configuration for each rat that illustrated the distribution spread of RMS values among the channels. The area under the curve (AUC) for each amplitude profile was calculated using trapezoidal numerical integration (MATLAB, *trapz*).

The AUC metric was chosen over alternative metrics such as spatial entropy or localization indices as the primary measure of spatial selectivity. It provides a single, continuous index of how concentrated or distributed muscle activation is across the array. Lower AUC values indicate that eEMG amplitude was concentrated among a few channels, reflecting greater selectivity, compared with higher AUC values. Higher AUC values indicate that eEMG amplitude was more evenly distributed across channels, reflecting less selectivity. Because AUC quantifies the degree of spatial concentration but not the anatomical location of activation, we used two complementary analyses to capture additional information about the spatial activation, identifying the location of peak activation, which was presented in histograms, and calculating pairwise spatial correlations (Pearson r) between electrode pairs implanted within the same fascicle to assess overlap in muscle activation patterns across LIFEs.

### 2.7. Statistical analyses

All statistical analyses were conducted using R (version 4.5.0, R Core Team, 2024), with statistical significance set at *α* ⩽ 0.05. Linear mixed-effects regression models with fixed and random effects detailed below were employed to address our research questions. These models were implemented using the *lme4* package [30] utilizing Restricted Maximum Likelihood Estimation. Where applicable, post hoc comparisons were performed using estimated marginal means calculated with the *emmeans* package [31], with adjustments for multiple comparisons (Tukey or Dunnett). Model assumptions, including linearity and normality of residuals, were evaluated to ensure the validity of model fitting. The model equations are presented in R syntax rather than in standard linear mixed model notation. Prior to fitting the statistical model, stimulation level values were mean-centered to facilitate interpretation of the regression coefficients. By including mean-centered stimulation level, the model intercept represented the eEMG amplitude response at the mean stimulation level, rather than at a stimulation level of zero.

#### 2.7.1. Effect of stimulation level on eEMG amplitude

To determine the effect of monopolar stimulation amplitude level on eEMG RMS amplitude across channels, we constructed a linear mixed-effects model with fixed effects of *Stimulation Amplitude_c_ (mean-centered), EMG Channel*, and the *Stimulation Amplitude_c_-by-EMG Channel* interaction. To fully account for statistical dependencies in the data due to its hierarchical structure, we included random intercepts for *Rat, Channel* nested within *Rat*, and *LIFE* nested within *Rat*, with a random slope for *Stimulation Amplitude_c_* by *LIFE* nested within *Rat*. 

\begin{align*}&amp; {\text{(Model 1):}} \,\, RMS\sim 1 + StimAmplitud{e_c}*Channel\nonumber\\ &amp;\quad + \left( {1{\mathrm{|}}Rat} \right) + {\text{ }}\left( {1{\mathrm{|}}Rat:Channel} \right)\nonumber\\ &amp;\quad + {\text{ }}(1 + StimAmplitud{e_c}|Rat:LIFE).\end{align*}

#### 2.7.2. Effect of stimulation from different LIFEs on eEMG amplitude

To evaluate the effect of monopolar stimulation delivered by different LIFEs at different stimulation levels, the model from the previous section (the effect of *Stimulation Amplitude_c_-by-EMG Channel* on eEMG RMS amplitude) was used again. Our experimental design precluded inclusion of *LIFE* as a fixed effect, given that LIFE placement was not anatomically standardized across animals (i.e. LIFE #1 in one rat was not implanted at a location equivalent to LIFE #1 in another rat). Therefore, we examined the influence of different LIFEs by comparing the model variances due to *LIFE, Stimulation Amplitude_c_, Rat*, and *Channel* in relation to the global average RMS amplitude. To do so, we extracted the corresponding variances of the random intercepts and slopes. This allowed us to evaluate which factor caused the most variation in eEMG amplitude (i.e. using different LIFEs, using different stimulation amplitudes, recording from different channels, or recording from different rats).

#### 2.7.3. Effect of stimulation level on the spatial distribution of eEMG amplitude

To determine the effect of stimulation level on the spatial distribution of eEMG (quantified as AUC of the eEMG amplitude profiles, we constructed a linear mixed effects model with fixed effects of *Stimulation Amplitude_c_,* random intercepts of *Rat* and *LIFE* nested within *Rat,* and a random slope of *Stimulation Amplitude_c_* by *LIFE* nested within *Rat.* After visualizing the AUC data vs. stimulation level, we also included polynomial terms in the model, with fixed effects of *Stimulation Amplitude_c_*^2^ and *Stimulation Amplitude_c_*^3^ and random slopes of the polynomial terms by *LIFE* nested within *Rat.*

\begin{align*}&amp; {\text{(Model 2):}} \,\, AUC\sim 1 + StimAmplitud{e_c} + StimAmplitude_c^2 \\ &amp;\quad + StimAmplitude_c^3 + \left( {1{\mathrm{|}}Rat} \right) + \left( 1 + StimAmplitud{e_c}\right.\\ &amp;\quad\left.+ StimAmplitude_c^2 + StimAmplitude_c^3{\mathrm{|}}Rat:LIFE \right)).\end{align*}

We also examined whether the shape and variability of the ranked amplitude profiles changed with stimulation level. At each stimulation level, we ensemble-averaged the ranked amplitude profiles from all LIFEs and rats. We characterized the shapes of the resulting average profiles using polynomial regression and by examining the coefficients across stimulation levels. The average amplitude profile for each stimulation level was fitted to a cubic polynomial using least squares in the following form:

\begin{align*}{\mathrm{RM}}{{\mathrm{S}}_{{\mathrm{Norm}}}}\left( x \right) = {\beta _0} + {\beta _1}x + {\beta _2}{x^2} + {\beta _3}{x^3}\end{align*}

where *β*_0_ is the intercept term, *β*_1_–*β*_3_ are the coefficients of the polynomial curve, and x is the rank of the channel (e.g. 1st, 2nd… rather than the channel number).

To examine the inter-animal variability of the ranked amplitude profiles at each stimulation level, we ensemble-averaged the amplitude profiles from all LIFEs for each rat and stimulation level. We then calculated the standard deviation of the mean profile across all sorted channels. This resulted in a standard deviation profile by sorted channel, the AUC of the standard deviation profile (*trapz*, MATLAB), and a calculated mean standard deviation among channels.

#### 2.7.4. Effect of stimulation type on eEMG amplitude

To evaluate the effect of stimulation type (monopolar or bipolar) on eEMG RMS amplitude across channels and at different stimulation levels, we constructed a linear mixed effects model with fixed effects of *Stimulation Type, Stimulation Amplitude_c_, EMG Channel*, and their three-way interaction.

To fully account for statistical dependencies within the data, we included random intercepts for *Stimulation Type* nested within *Rat, Channel* nested within *Rat*, and a random slope for *Stimulation Amplitude_c_* by *Stimulation Type* nested within *Rat*. A random intercept of *Rat* was included initially but then removed because it accounted for zero variance and created a model singularity.

\begin{align*}&amp;{\text{(Model 3):}}\,\, RMS\sim 1 + {\text{ }}StimType*Channel\\ &amp;\quad *StimAmplitud{e_c} + \left( {1{\mathrm{|}}Rat:Channel} \right) \\ &amp;\quad + \left( {1 + StimAmplitud{e_c}{\mathrm{|}}Rat:StimType} \right).\end{align*}

#### 2.7.5. Effect of stimulation type on eEMG spatial distribution

We modeled stimulation type as having three levels: bipolar stimulation with two LIFEs, monopolar stimulation with one of the two LIFEs (labeled *MS1*), and monopolar stimulation with the other of the two LIFEs (labeled *MS2*). To determine whether the spatial distribution of the eEMG (quantified as the AUC of the eEMG amplitude profiles) differed among these three stimulation configurations and stimulation levels, we constructed a linear mixed effects model with fixed main effects of *Stimulation Type, Stimulation Amplitude_c_, and* their two-way interaction. We included a random intercept for *Rat* and a random slope for *Stimulation Amplitude_c_* by *Stimulation Type* nested within *Rat*. To be consistent with Model 2 above, we also included polynomial terms in the model, with fixed effects of *Stimulation Amplitude_c_*^2^ and *Stimulation Amplitude_c_*^3^ and random slopes of the polynomial terms by *Stimulation Type* nested within *Rat.*

\begin{align*}&amp; {\text{(Model 4):}}\,\, AUC\sim 1 + StimType*StimAmplitud{e_c} \\ &amp;\quad+ StimType*StimAmplitude_c^2 + Stim{\text{ }}Type\\ &amp;\quad *StimAmplitude_c^3 + \left( {1{\mathrm{|}}Rat} \right) + ( 1 + StimAmplitud{e_c}\\ &amp;\quad + StimAmplitude_c^2 + StimAmplitude_c^3{\mathrm{|}}Rat:StimType ).\end{align*}

#### 2.7.6. Effect of stimulation level on the HD-eEMG channel with the highest eEMG amplitude

For each stimulation level, the channel with the highest RMS value for each LIFE was identified, and the number of occurrences per channel was counted to build histograms and perform the analyses. Analyses were performed separately for the two types of stimulation (monopolar and bipolar) at each stimulation level.

#### 2.7.7. Effect of electrode placement and stimulation level on spatial correlation of eEMG activity elicited by LIFEs

To quantify overlap between muscle activation patterns produced by different LIFEs, pairwise spatial correlation (Pearson *r*) was calculated between electrode pairs within the same fascicle at the same stimulation level. Because stimulation levels did not align exactly across LIFEs, normalized RMS values for each channel were interpolated onto a common set of stimulation levels (xTH), restricted to each LIFE’s tested range without extrapolation.

The normalized RMS responses at each of the 26 HD-eEMG channels elicited by each electrode in the pair were compared on a channel-by-channel basis (i.e. For Rat ‘5’, Channel 1 of LIFE 1 with Channel 1 of LIFE 2 at 1.2xTH) and a single Pearson correlation coefficient was computed across all 26 paired values, resulting in one correlation coefficient per electrode pair per stimulation level. This was repeated across all stimulation levels to assess whether spatial activation patterns between electrode pairs varied with amplitude. Comparisons at a given stimulation level were excluded if, for both LIFEs, every EMG Channel’s RMS value fell below 5% of that LIFE’s own maximum RMS, to avoid computing correlations on signals that were predominantly noise.

## 3. Results

We recorded M-wave responses from an HD-eEMG grid placed on the exposed gastrocnemius that resulted from intrafascicular stimulation of the sciatic nerve using a monopolar stimulation configuration (49 LIFEs across 17 different rats) and a bipolar stimulation configuration (27 pairs of LIFEs in a subset of nine rats).

### 3.1. Increasing monopolar stimulation amplitude induces graded muscle activation

**Figure 2.**
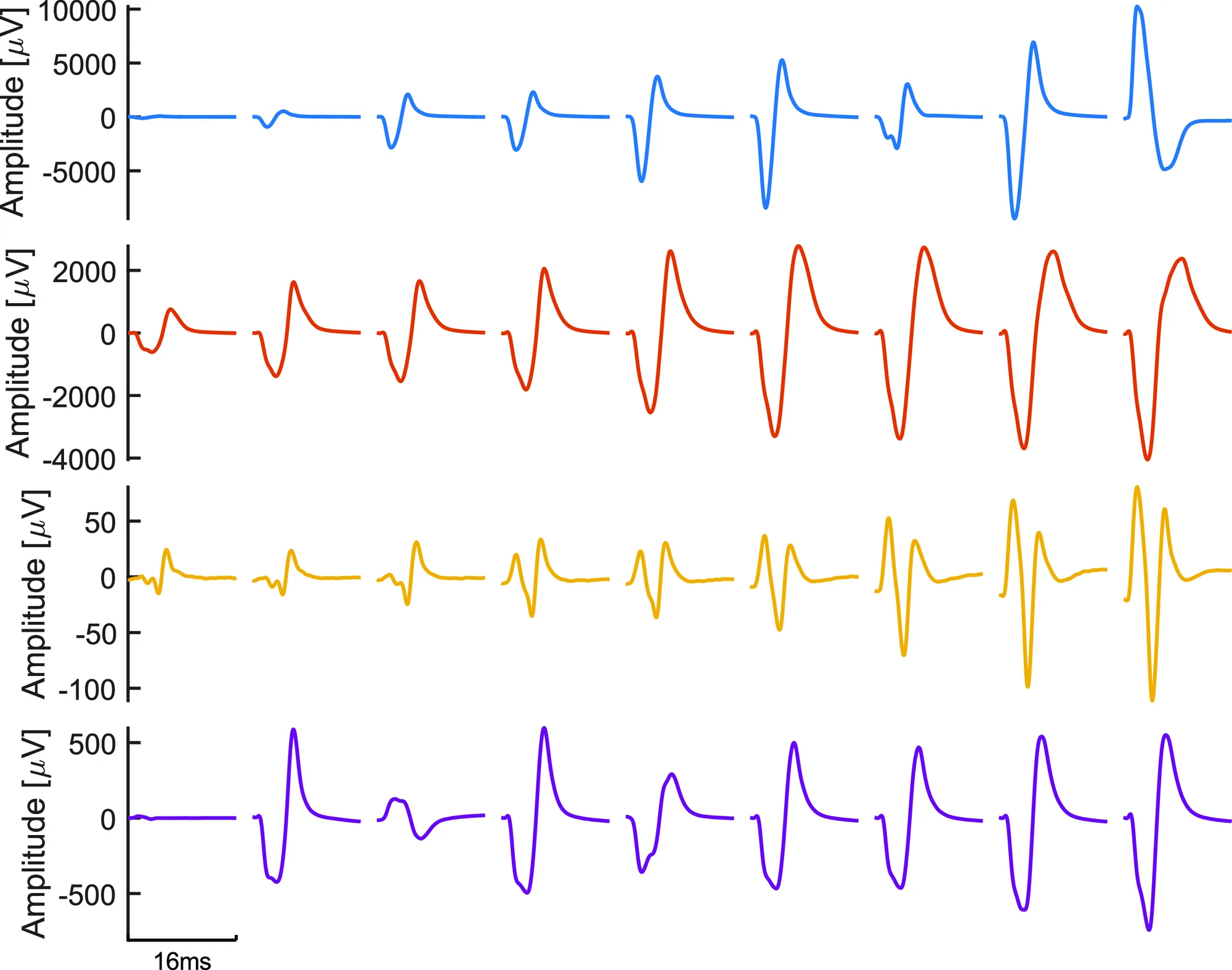
*Comparison of STA M-wave responses to monopolar stimulation using four LIFEs across the amplitude sweep.* The effect of increasing stimulation level on M-wave responses recorded from one HD-eEMG channel for four different LIFEs are shown. Each trace for a given row is the STA response amplitude (*y-axis*) over time (*x*-axis—16 ms each segment) for nine stimulation levels (increasing in amplitude from left to right), which collectively illustrate graded activation. Stimulation amplitudes are normalized to the activation threshold for each individual LIFE. The starting stimulation amplitude was set below this threshold to capture the progressive increase in neural recruitment.

Figure 2 shows M-wave responses in one HD-eEMG channel resulting from increasing stimulation amplitude using four different LIFEs. There was a significant main effect of *Stimulation Amplitude* (*F*(1,48) = 39, *p*< 0.0001) and a significant *Stimulation Amplitude-by-Channel* interaction (*F*(25 174 59) = 26, *p* < 0.0001), indicating that the effect of increasing stimulation level on RMS amplitude differed among the HD-eEMG channels.

Monopolar stimulation generally resulted in graded muscle activation that increased with increasing stimulation amplitude. The nature of the graded activation differed slightly depending on the LIFE used. Stimulation with LIFE #1 elicited subtle changes in M-wave shape at the initial stimulation level, with more pronounced responses after the fourth stimulation level. The increase in M-wave amplitude was generally monotonic, with one abrupt reduction at the seventh stimulation level. The highest stimulation level resulted in the reversal of the M-wave polarity. Stimulation with LIFE #2 and LIFE #3 showed more consistent graded increases in response magnitude across stimulation levels. Finally, stimulation with LIFE #4 demonstrated a less consistent increase at lower stimulation levels, with a subtle but consistent increase in response magnitude at the five highest stimulation levels.

### 3.2. Stimulation using different LIFEs implanted in the same fascicle produces variable response magnitudes and different spatial patterns of muscle activity

#### 3.2.1. Effects of different LIFEs on muscle response magnitude

The model estimated the global mean RMS value of the M-wave responses across rats, LIFEs, eEMG channels, and stimulation amplitudes was 428.7 *µ*V. Table 1 shows the standard deviations in RMS amplitude attributed to variability among rats (random intercept of *Rat*), among EMG channels within each rat (random intercept of *Channel* nested within *Rat*), among LIFEs within each rat (random intercept of *LIFE* nested within *Rat*), and due to the effect of stimulation amplitude varying by LIFE (random slope of *Stimulation Amplitude* by *LIFE* nested within *Rat*).

**Table 1.** Fixed effect coefficients and the standard deviation attributed to each random effect from the *Stimulation Amplitude-by-EMG* Channel model for RMS amplitude.

Fixed effect		Coefficient
	*Intercept*	428.7

	*Stimulation Amplitude*	1298.1

Random effect		Std. Dev.

Intercept	*Rat*	227.6
	*Channel* nested within *Rat*	194.1
	*LIFE* nested within *Rat*	414.4

Slope	*Stimulation Amplitude by* *LIFE* nested within *Rat*	1295.7

	Residual	339.9

The random intercept of *LIFE* nested within *Rat* contributed the greatest variability to RMS amplitude, with a standard deviation of 414.4 *µ*V. This indicates that while the mean RMS amplitude was 428.7 *µ*V, for any given LIFE, the RMS amplitude could vary around 428.7 with a standard deviation of ± 414.4 *µ*V. The standard deviations in the RMS amplitude due to *Rat* and *Channel* were lower, at 227.6 and 194.1 *µ*V, respectively. The substantial variability in RMS amplitudes from using different LIFEs demonstrates their flexibility and potential for further development of targeted selectivity.

The effect of *Stimulation Amplitude_c_* also varied substantially among LIFEs, indicating that the rate of RMS amplitude increase with increasing stimulation level is highly dependent on the specific LIFE used for stimulation. The model estimate for the effect of *Stimulation Amplitude_c_* (on average among rats, LIFEs, and channels) was 1298.1 *µ*V xTH^−1^, indicating the amount that RMS amplitude would increase with a 1x threshold increase in stimulation level. However, the variability of this effect varied substantially by LIFE, with a standard deviation of 1295.7 *µ*V xTH^−1^. This indicates that for the middle 68% of a population of LIFEs, the increase in RMS amplitude with a 1x threshold increase in stimulation level would range from 2.4 to 2593.8 *µ*V/ xTH.

#### 3.2.2. Effects of different LIFEs and stimulation amplitude on spatial muscle activation patterns

**Figure 3.**
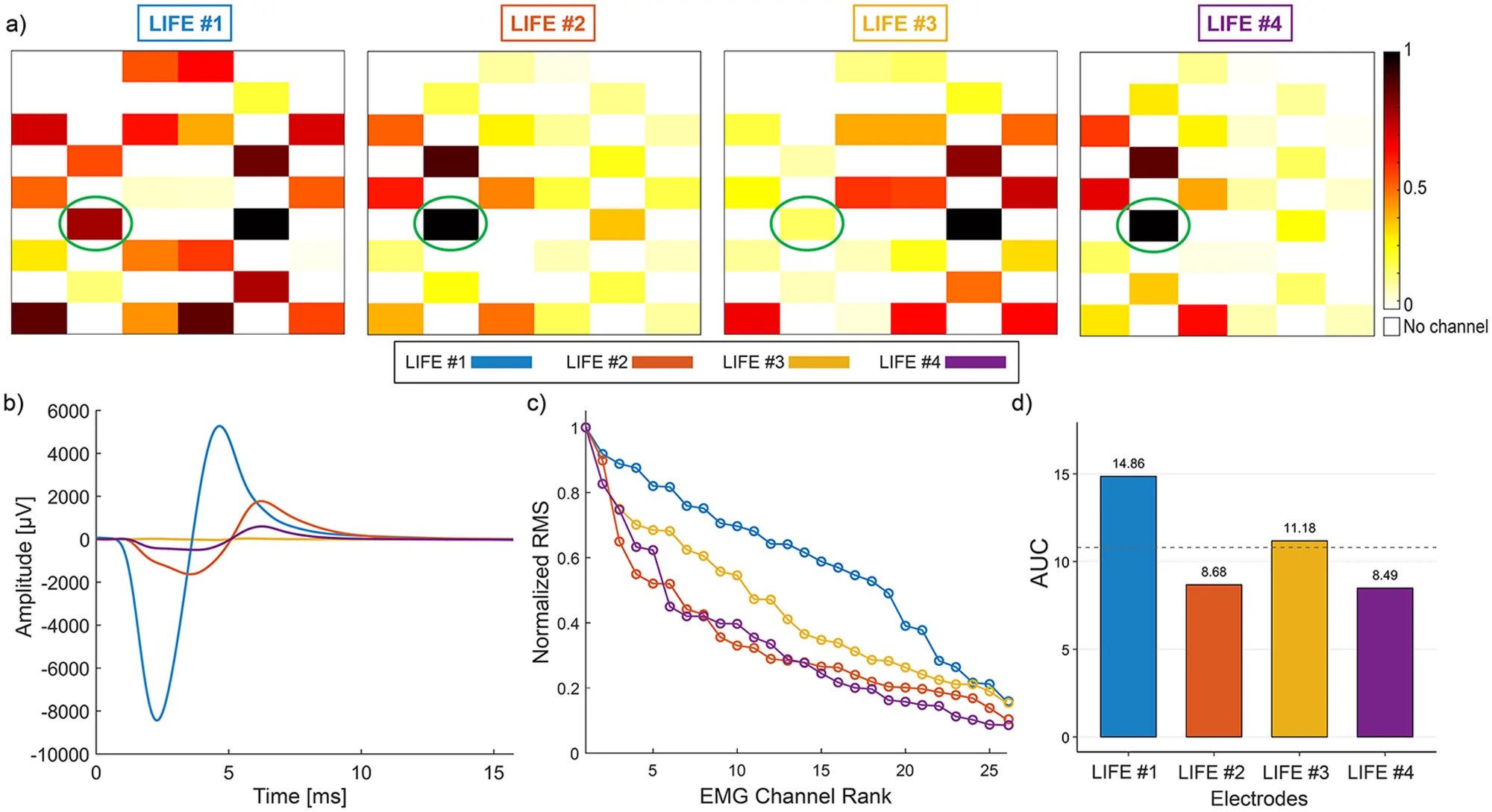
*Comparison of spatial muscle activation resulting from monopolar stimulation configurations.* (a) Heatmaps showing eEMG amplitude across channels for stimulation using four different LIFEs (LIFE #1—#4) in one rat at one stimulation level (1.2xTH), with color representing RMS amplitude, normalized to the highest value across all eEMG channels for each grid. Green circles highlight the spatial location of the highest muscle activation obtained across the grid when using LIFE #2 or LIFE #4, demonstrating that each LIFE evokes distinct muscle activation patterns. (b) STA M-wave responses for each LIFE at the same stimulation level (xTH) showing different magnitude and waveform shapes. (c) Ranked normalized RMS profile curves across EMG channels, demonstrating that stimulation using LIFE #1 elicited the broadest activation with low selectivity, while use of LIFE #2 and LIFE #4 exhibited higher selectivity with lower diffusivity of activation. (d) Bar plots showing the calculated AUC from the ranked RMS profile curves.

RMS amplitude heatmaps demonstrated pronounced differences in the spatial distribution of muscle activation among the LIFEs, including the channel that exhibited the highest amplitude. Representative data from one rat are shown in figure 3(a). Among the LIFEs used, the activation patterns produced by LIFE #2 and LIFE #4 were the most similar, exhibiting a peak activation along the left side of the grid. Conversely, the use of LIFE #3 produced a markedly distinct pattern with peak activation observed on the right side of the grid. Stimulation with LIFE #1 resulted in a broader activation pattern across the entire grid. These differences in the spatial distribution of eEMG are consistent with the statistical analysis of group data above showing that different LIFEs contribute the most variability to mean RMS level.

Additionally, M-waves had distinctly different waveform shapes depending on which LIFE was utilized for stimulation. Figure 3(b) shows M-wave responses from stimulation with four different LIFEs in a single HD-eEMG array channel (circled in green in figure 3(a)). This observation supports the possibility that stimulation with each LIFE recruited a different combination of nerve fibers.

The ranked eEMG amplitude profiles corresponding to the data in figures 3(a) and (b) are shown in figure 3(c), with their respective AUC values shown in figure 3(d). In alignment with visual assessment of the RMS value heatmaps, stimulation with LIFE #1 demonstrated the least selective recruitment, evidenced by the flattest profile curve and the highest AUC. In contrast, LIFE #2 and LIFE #4 exhibited profile curves with steeper decline and lower AUC values, reflecting a more spatially focused activation concentrated within fewer eEMG channels. Stimulation using LIFE #3 showed an intermediate profile, occupying a position between these two extremes.

For group data, the model estimated global mean AUC of the ranked eEMG profiles across rats, LIFEs, and stimulation levels was 12.48. Table 2 shows the standard deviations in the fixed effect coefficients attributed to variability among rats (random intercept of *Rat*), among LIFEs within each rat (random intercept of *LIFE* nested within *Rat*), and due to the effect of stimulation amplitude varying by LIFE (random slope of *Stimulation Amplitude* by *LIFE* nested within *Rat*). The global mean AUC varied by LIFE with a standard deviation of 2.08, which was almost two times its standard deviation due to rat (1.15).

**Table 2.** Fixed effect coefficients and the standard deviation attributed to each random effect from the *Stimulation Amplitude* model for AUC.

Fixed effect		Coefficient
	*Intercept*	12.48
	*Stimulation Amplitude*	2.23

Random effect		Std. Dev.

Intercept	*Rat*	1.15
	*LIFE* nested within *Rat*	2.08

Slope	*Stimulation Amplitude by* *LIFE* nested within *Rat*	4.87
	*Stimulation Amplitude^2^ by* *LIFE* nested within *Rat*	13.24
	*Stimulation Amplitude^3^ by* *LIFE* nested within *Rat*	23.72

	Residual	1.31

**Figure 4.**
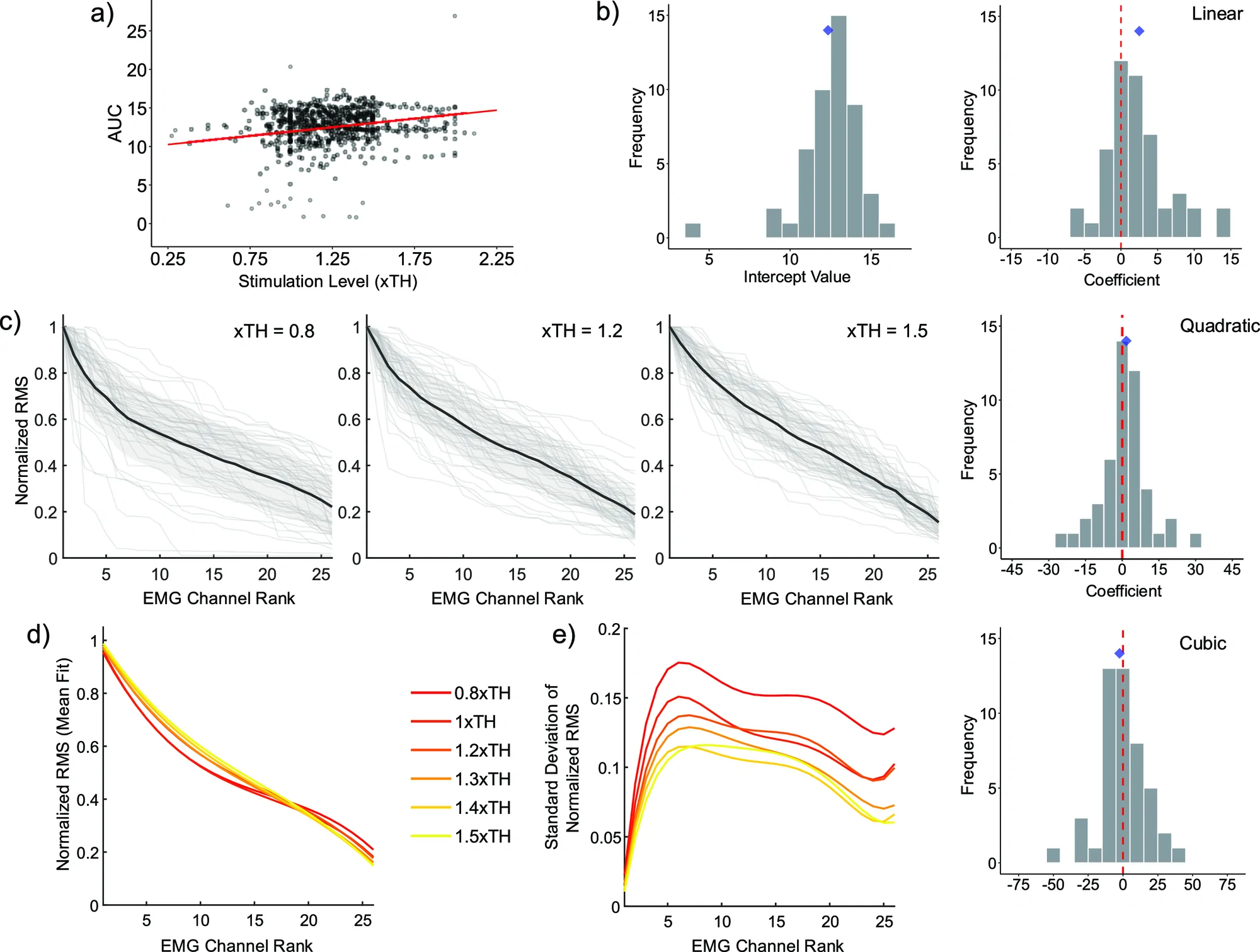
*The effect of monopolar stimulation using different LIFEs across stimulation levels.* (a) AUC vs stimulation level (xTH) across all LIFEs and rats with the regression line shown in red reflects the significant fixed effect of stimulation amplitude and model intercept. (b) Histograms of the intercept, linear, quadratic, and cubic coefficients for all LIFEs across rats, fixed effect coefficient shown as blue diamond. (c) Ranked normalized RMS profile curves of all electrodes (gray) at three different stimulation levels: subthreshold (0.8xTH), slightly above threshold (1.2xTH) and highest stimulation level (1.5xTH). The average profile is shown in black with its standard deviation in shaded gray. (d) Polynomial curves fit to the average ranked RMS profiles for each stimulation amplitude bin. (e) The standard deviation around the average ranked eEMG amplitude as a function of stimulation levels (xTH).

Figure 4 shows how stimulation level affects the AUC (a), (b), shape (c), (d), and variability (e) of ranked eEMG amplitude profiles figure 4(a) shows AUC vs. stimulation level for data from all LIFEs and rats. The regression line shown in red reflects the significant fixed effect of stimulation amplitude and model intercept. The effect of stimulation amplitude on AUC was significant (2.23/xTH; *F*(1,50) = 7.66, *p* = 0.0079) but the quadratic and cubic effects of stimulation amplitude were not (quadratic term: 2.09/xTH^2^; *F*(1,29) = 0.35, *p* = 0.35; cubic term: −2.04/xTH^3^; *F*(1,32) = 0.25, *p* = 0.62) (Model 2). Visual inspection of figure 4(a) gives the impression of an overall non-linear relationship, which would be in contrast to the statistical model results. However, the statistical model appropriately accounts for the fact that the data points are not independent, which visual inspection of this plot cannot do. For example, all of the data points in the lower left corner below an xTH value of 0.7 are from two LIFEs within the same rat. When accounting within the linear mixed effects model for the correlation between data points for each individual LIFE, the correlation between data points for each individual rat, and the unequal number of data points across LIFEs and rats, the non-linear fixed effect (i.e. across the entire population) coefficients are not significantly different from zero.

The overall effect of stimulation amplitude on AUC (on average across rats and LIFEs) was relatively small in magnitude (2.23/xTH). In comparison, the standard deviations of the effects of stimulation amplitude by LIFE, stimulation amplitude^2^ by LIFE, and stimulation amplitude^3^ by LIFE were substantial (table 2: 4.87/xTH, 13.24/xTH^2^, 23.72/xTH^3^). Histograms of the linear, quadratic, and cubic coefficients for all LIFEs are shown in figure 4(b) and illustrate this variability relative to zero and to the corresponding fixed effect coefficients shown as blue diamonds. Accordingly, pseudo-*R*^2^ for the fixed effect of stimulation amplitude was 0.03, indicating that it accounted for a nearly negligible proportion of the variance in AUC. In contrast, the total pseudo-*R*^2^ (i.e. including both fixed and random effects) was 0.86, demonstrating that the random effects representing inter-animal and inter-LIFE variability accounted for the majority of the variance in AUC. Taken together, these findings demonstrate that increasing stimulation amplitude resulted in changes in AUC that occurred in a wide variety of patterns, both linear and non-linear, among LIFEs.

Figure 4(c) shows the ranked eEMG amplitude profiles derived from the full dataset at three different stimulation amplitudes. Individual profiles for each LIFE used are depicted in gray, whereas the mean profile and its standard deviation are shown in black and shaded gray, respectively. The overall shape of the profiles gradually changed across the stimulation levels, becoming progressively flatter and more linear as the stimulation amplitude increased. The coefficients obtained from the polynomial fit of the mean profile for each stimulation level are listed in table 3. As the stimulation amplitude increased, both the negative cubic and linear coefficients exhibited an upward trend, the positive quadratic coefficient exhibited a downward trend, and the intercept remained stable. This systematic pattern is consistent with broader activation of the muscle and reduced selectivity with increasing stimulation amplitude.

**Table 3.** Coefficient of polynomial fit for profile curves at each stimulation level (monopolar stimulation).

xTH	*β*_3_ (*x* 10^−5^)	*β*_2_ (*x* 10^−3^)	*β*_1_ (*x* 10^−2^)	*β*_0_
0.8	−9.0	4.5	−8.7	1.04
1	−8.4	4.1	−8.4	1.03
1.2	−6.7	3.3	−7.4	1.05
1.3	−6.0	3.0	−7.0	1.04
1.4	−5.5	2.7	−6.7	1.04
1.5	−5.6	2.6	−6.6	1.05

In addition to the changes in the average profile shape as a function of stimulation intensity (figure 4(d)), notable differences in the variability of the individual profiles were also apparent (figure 4(e)). At sub-threshold stimulation levels (0.8 xTH), individual profile curves displayed considerable variability among the LIFEs used. This variability qualitatively decreased at 1.2 xTH and reached its minimum at 1.5 xTH. This pattern indicated a more consistent pattern of muscle activation across the electrodes and animals as the stimulation amplitude increased.

**Figure 5.**
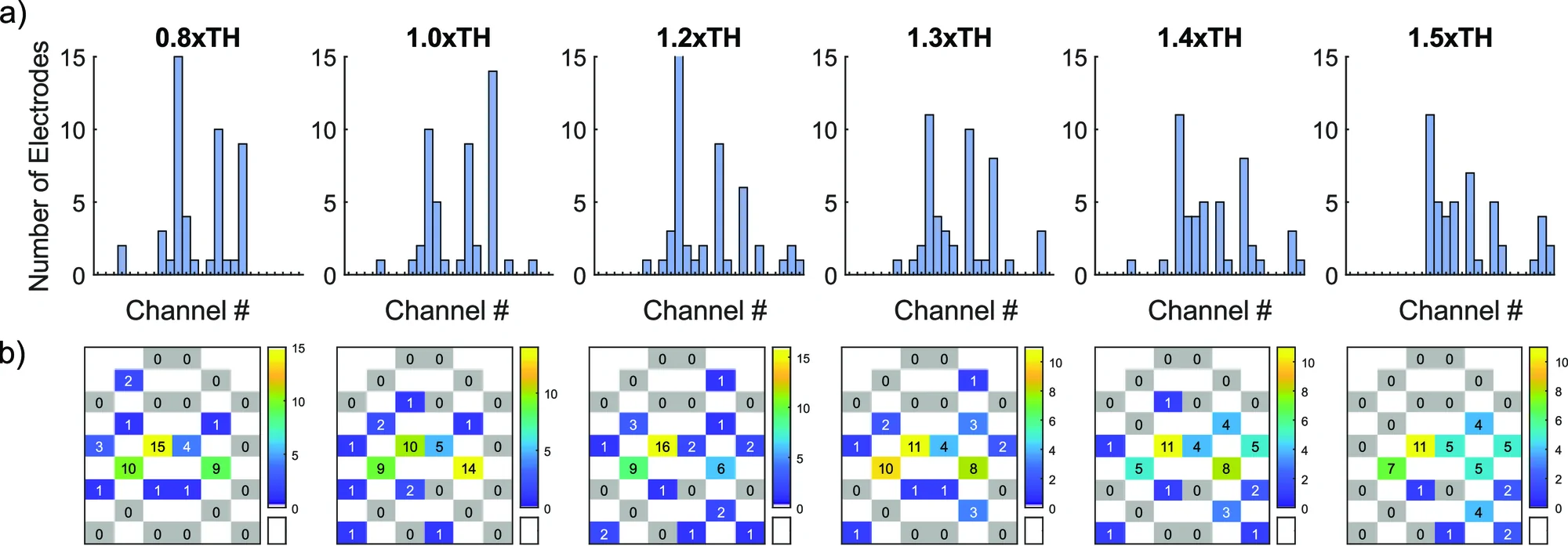
*Assessment of distribution of highest value EMG channel across all LIFEs.* (a) Histograms showing the EMG channel with the highest value of the grid across different stimulation levels (xTH). (b) Heatmaps from the histograms in (a) showing the spatial distribution of the most activated EMG channel position on the grid across all stimulation levels. A concentrated heatmap indicates a consistent highest EMG channel position, whereas a diffuse distribution reflects variability in the location of peak activation on the muscle across stimulation levels.

We also cataloged the channel with the maximum RMS amplitude for each LIFE used, and the stimulation amplitude condition for all conditions. Figure 5(a) shows histograms of the frequency of occurrence for each channel. For all stimulation amplitudes, the frequency distribution of channels with the maximum RMS value was not normal, with multiple peaks (figure 5(a)), indicating a non-random spatial organization of muscle activation. Instead, the distributions were concentrated in a limited set of channels that were predominantly located near the center of the grid. They were also clustered into distinct regions, showing off-center peaks (figure 5(b)). Although increasing the stimulation amplitude produced modest shifts in the relative frequency of occurrence, the locations of the peaks generally remained stable (channels 8, 6, and 21).

**Figure 6.**
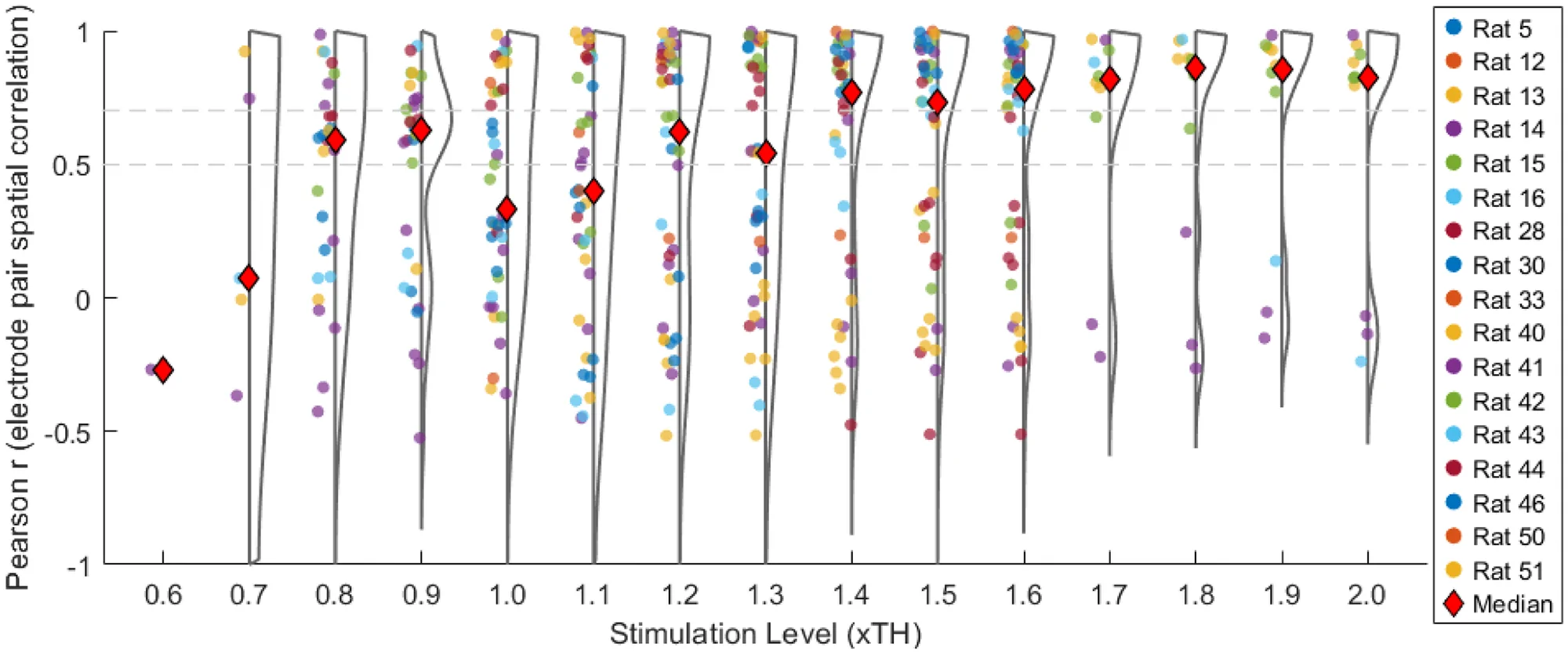
*Spatial correlation between activity elicited by LIFE pairs.* Pairwise spatial correlation between muscle activation patterns elicited by monopolar stimulation from pairs of LIFEs within the same fascicle, across different stimulation levels, indicate that muscle activation patterns becoming progressively similar as stimulation amplitude is increased. Individual points represent single electrode pair comparisons; color indicates the rat. Gray curves show the distribution of correlation values at each stimulation level. Red diamonds represent the median correlation. Dashed horizontal lines at *r* =0.5 and *r* = 0.7 denote the boundaries between low, intermediate and high spatial correlation categories.

Pairwise spatial correlation between activation patterns elicited by LIFEs was also assessed at each stimulation level, as illustrated in figure 6. At subthreshold levels, below 0.8xTH, comparisons were limited (*n* = 1 at 0.6xTH, *n* =5 at 0.7xTH, for only 1–2 rats) and they were predominantly low correlation (100% at 0.6xTH, 60% at 0.7xtH), consistent with different muscle pattern activation, but with a small sample size. Near threshold, from 0.8 to 0.9xTH, low and high correlation pairs have similar percentages (26%–41% low, 26%–31% high). From 1 to 1.3xTH, low correlation pairs are more common (44%–56%) than high correlation pairs (29%–44%), indicating limited overlap of activation patterns in this range. From 1.4 to 2xTH, this trend is reversed, with more high correlation pairs (53%–70%) than low correlation pairs (20%–40%), indicating that the overlap between LIFE pairs increased with the stimulation amplitude and remained high at the highest stimulation levels. This pattern is consistent with the notion that an increase in stimulation level would activate a larger portion of the fascicular cross-section, thus producing more overlap of activation across the electrode pair. Overall, these results indicate that different LIFEs and different electrode placements produce distinct activation patterns consistently near and just above threshold, with muscle activation patterns becoming progressively similar as stimulation amplitude is increased.

#### 3.2.3. Bipolar and monopolar stimulation produce graded but different muscle activation patterns

**Figure 7.**
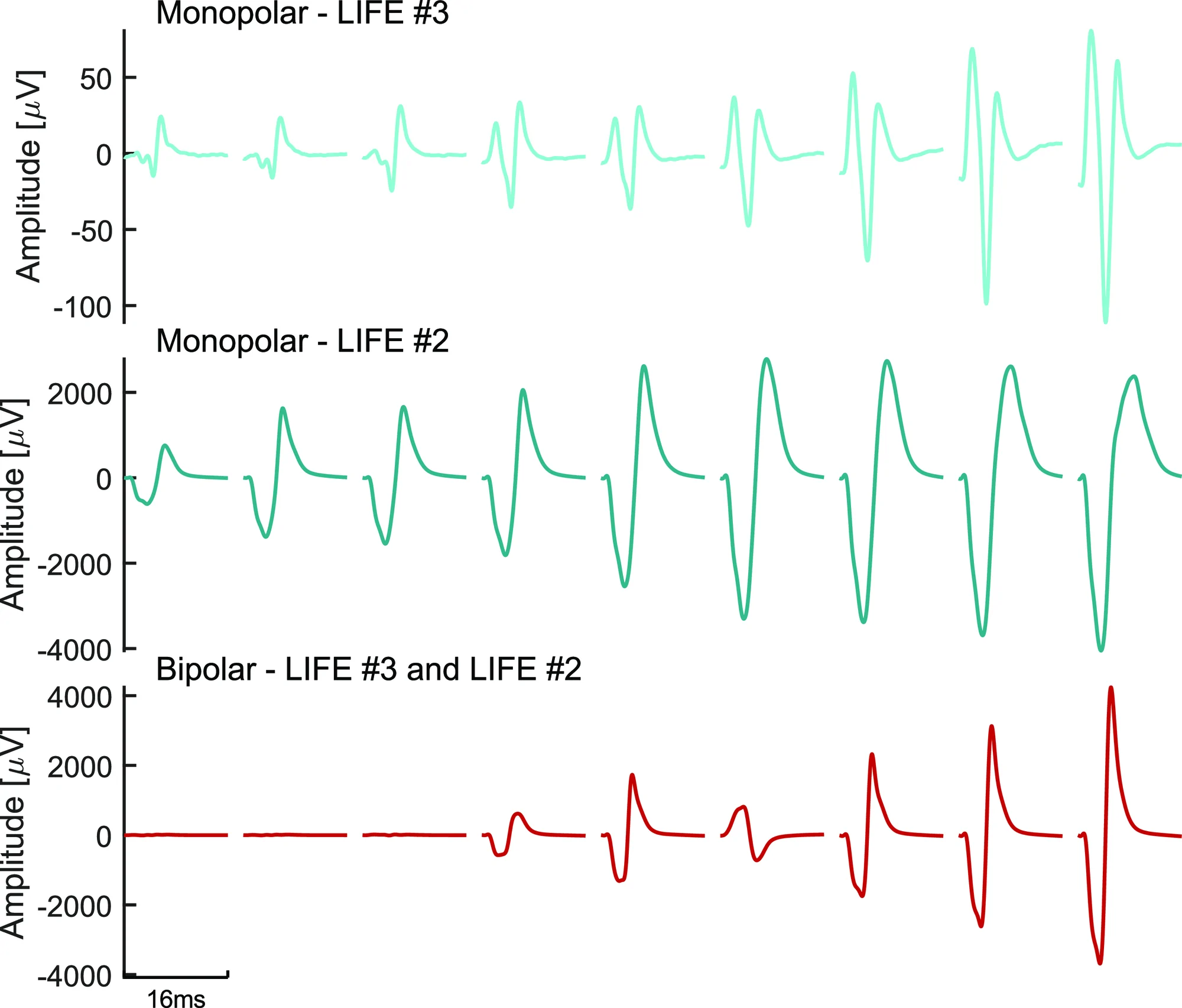
*Comparison of graded M-wave responses across monopolar and bipolar stimulation configurations.* The magnitude of evoked M-wave responses increased with increasing stimulation amplitude. Top and middle rows: example responses from one rat on monopolar stimulation using LIFE #3 (light blue; top) and LIFE#2 (dark blue; middle). Bottom row: example responses on bipolar stimulation using both LIFE #3 and LIFE #2 (red). Each trace for a given row is the stimulus-triggered average response amplitude (*y*-axis) over time (*x*-axis of 16 ms width) for nine stimulation levels (increasing in amplitude from left to right) and illustrates graded activation.

Figure 7 presents a comparison of the graded M-wave responses across monopolar and bipolar stimulation configurations. The top and middle rows illustrate the responses obtained from monopolar stimulation using two distinct LIFEs, designated Monopolar—LIFE #3 and Monopolar—LIFE #2 respectively. The bottom row depicts the responses from bipolar stimulation in which the same two LIFEs were utilized in a paired configuration. Across both monopolar and bipolar stimulation configurations, the magnitude of the evoked responses increased as the stimulation amplitude increased. This demonstrates that both stimulation configurations exhibit a graded recruitment of muscle fibers, with higher amplitudes producing greater M-wave responses.

For RMS amplitude, there was a significant *Stimulation Type* x *EMG Channel* interaction (*F*(50, 9717) = 8.6), *p* < 0.0001), indicating that the spatial distribution of EMG differed among the three stimulation conditions (bipolar stimulation and monopolar stimulation with the two constituent LIFEs used with bipolar stimulation).

**Figure 8.**
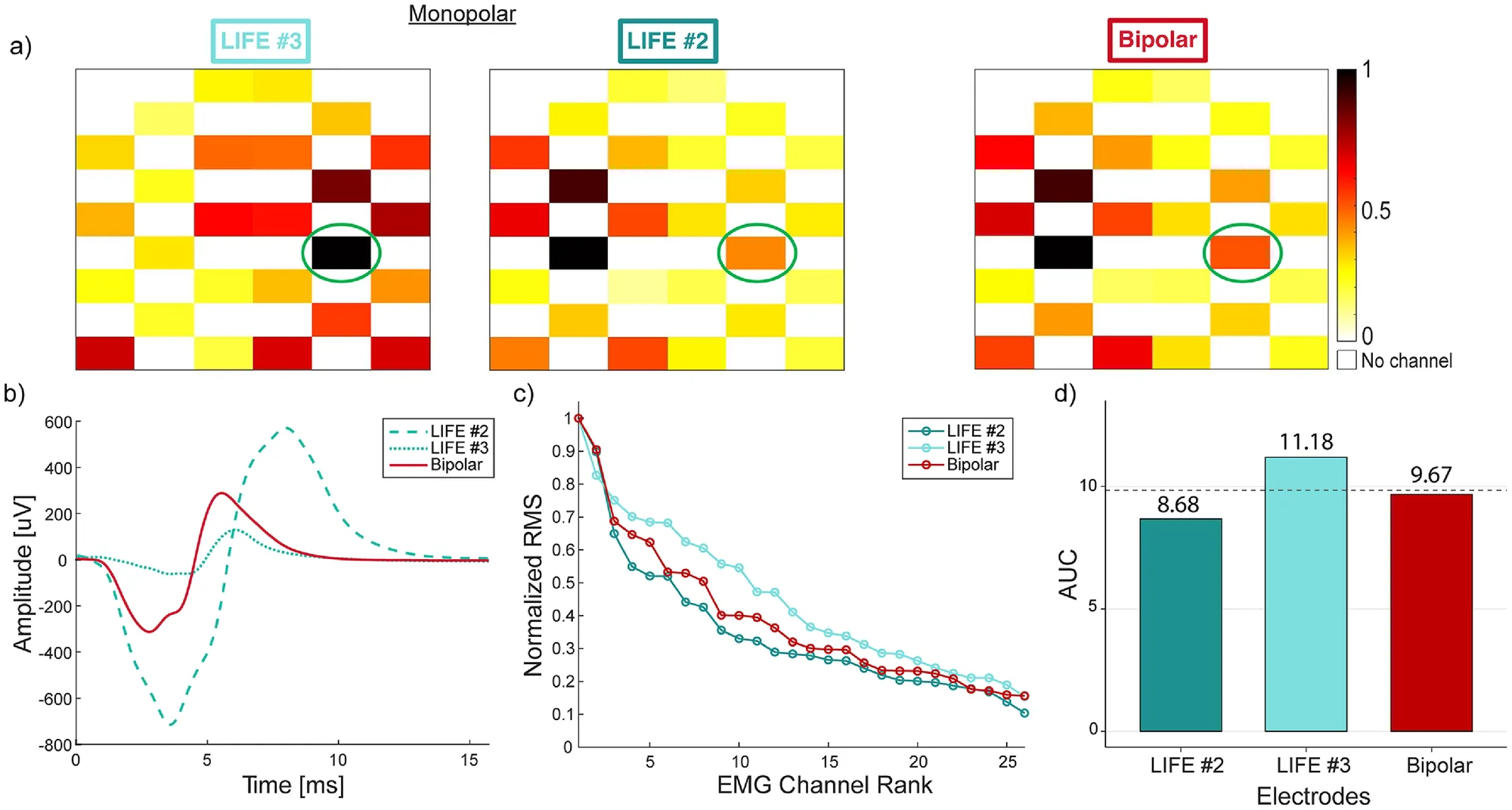
*Comparison of spatial HD-eEMG elicited on monopolar vs. bipolar stimulation configurations*. Data from a representative rat. (a) Heatmaps show HD-eEMG for all channels resulting from monopolar stimulation with two separate LIFEs (monopolar—LIFE #3 (left), monopolar—LIFE #2 (right)) and bipolar stimulation, with color representing normalized root mean square (RMS) amplitude. Green circles highlight the spatial location of the highest muscle activation for stimulation using LIFE #3. (b) Representative stimulus triggered average M-wave response from the HD-eEMG channel circled in green in (a), for the two monopolar and the bipolar stimulation configurations. Each stimulation was performed at the same stimulation level (xTH) but resulted in different response magnitudes and waveform shapes. (c) Ranked RMS profile curves for the two monopolar and the bipolar stimulation configurations. (d) Area under the curve (AUC) from the ranked RMS profile curves, with higher values indicating broader spatial activation and lower selectivity.

This group effect can be visualized through the representative example from one rat in figure 8. Heatmaps showing the RMS amplitude response to stimulation are shown in figure 8(a), M-wave responses are shown in figure 8(b), ranked eEMG amplitude profiles are shown in figure 8(c), and AUC values are shown in figure 8(d). Each of these outcomes illustrates differences in the response to bipolar stimulation with two LIFEs compared to responses from separate monopolar stimulations with the same LIFEs. In this example, the spatial activation pattern generated using LIFE #2 in a monopolar configuration closely resembled that observed with bipolar stimulation, whereas monopolar stimulation using LIFE #3 produced a significantly different, nearly inverted muscle activation pattern. The ranked eEMG amplitude profiles showed that monopolar stimulation using LIFE #2 yielded the steepest curve and the lowest AUC, indicating the highest selectivity among the three configurations. Bipolar stimulation produced a similar profile, although with slightly reduced selectivity, whereas stimulation using LIFE #3 resulted in the broadest spatial activation.

#### 3.2.4. Effect of increasing stimulation amplitude on spatial EMG with bipolar vs. monopolar stimulation

The *StimulationAmplitude_c_* x *StimulationType* x *Channel* interaction was significant (*F*(50, 9694) = 14.3), *p* < 0.0001), indicating that the effect of stimulation type on the amplitude and location of eEMG among channels depended on stimulation amplitude.

**Figure 9.**
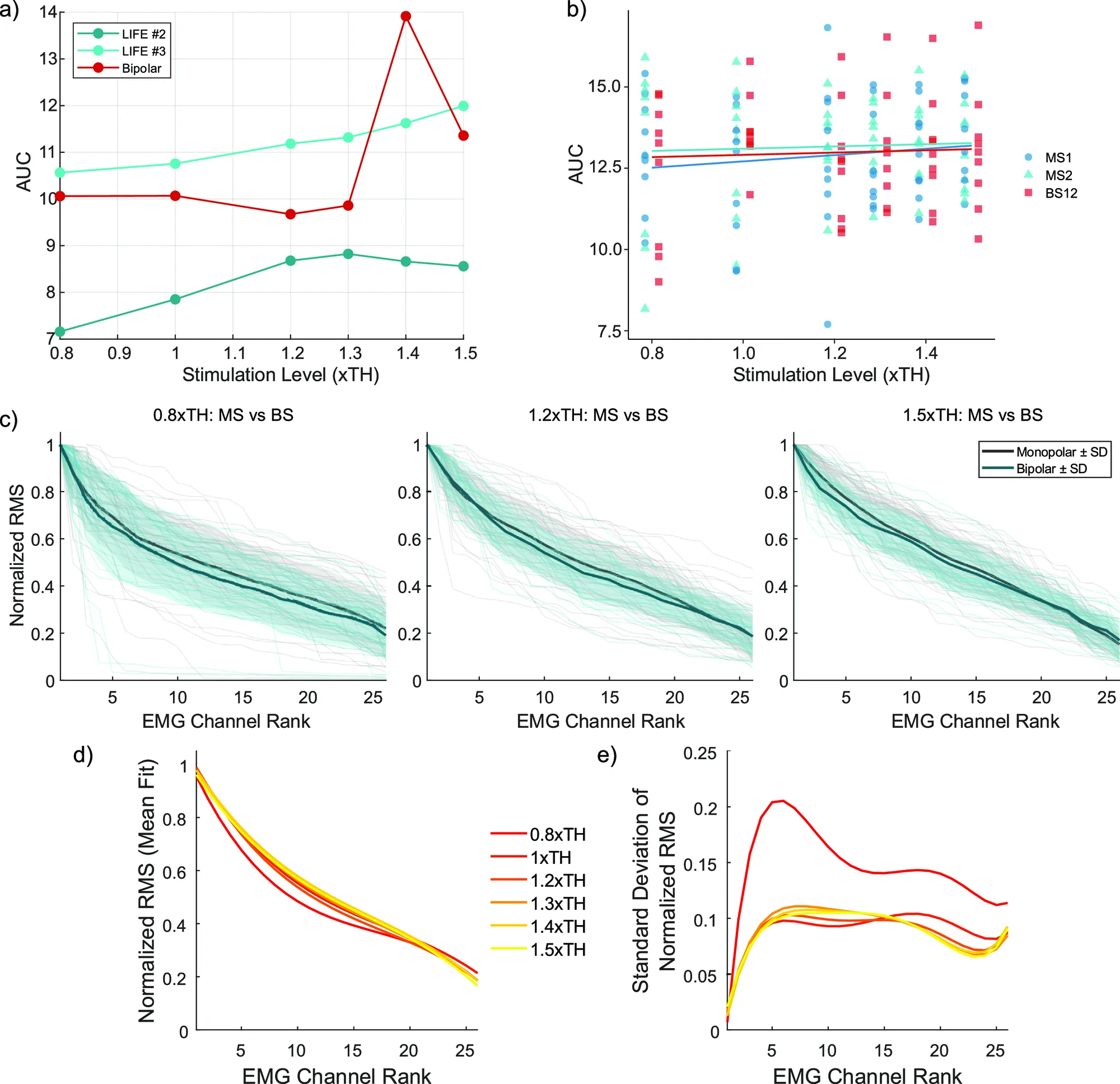
A*ssessment of intrafascicular selectivity for monopolar and bipolar stimulation configurations across stimulation levels.* (a) Example showing the area under the curve (AUC) for stimulation using two LIFEs in monopolar configuration and when used together in a bipolar configuration across different stimulation levels (same LIFEs as in figure 6). (b) AUC of ranked eEMG amplitude profiles across all LIFEs and animals for each stimulation level and stimulation type (monopolar configuration: Blue, bipolar configuration: Red). (c) Ranked eEMG amplitude profiles of all electrodes in monopolar configuration and pairs of electrodes in bipolar stimulation at three different stimulation levels: subthreshold (0.8xTH), slightly above threshold (1.2xTH) and high stimulation level (1.5xTH). (d) Curves fitted to the mean normalized root mean square (RMS) for bipolar stimulation across all the electrodes and stimulation levels. (e) Standard deviation of normalized RMS across ranked EMG channels for bipolar stimulation as a function of the stimulation level (xTH).

Examining the size of the spread of eEMG using the AUC metric (figures 9(a) and (b)), however, there were no significant effects of *StimulationType* (*p*= 0.77; estimated means: monopolar MS1: 12.9, monopolar MS2: 13.2, bipolar: 13.0), or its interactions with the polynomial effects of *StimulationAmplitude_c_* (linear: *p* = 0.16, quadratic: *p* = 0.90, cubic: *p* = 0.19) (figure 9(b)).

In addition, the average ranked eEMG profiles for the monopolar and bipolar configurations showed no major qualitative differences in overall shape, steepness, or variability for any of the stimulation levels (figures 9(c)–(e)). The coefficients of the polynomial fit for bipolar stimulation (not reported for brevity) were virtually identical to those for monopolar stimulation (table 3).

These findings indicate that while the different stimulation types altered the location of the eEMG and the effect of the stimulation amplitude on each channel, the size of the spread of the eEMG was stable.

**Figure 10.**
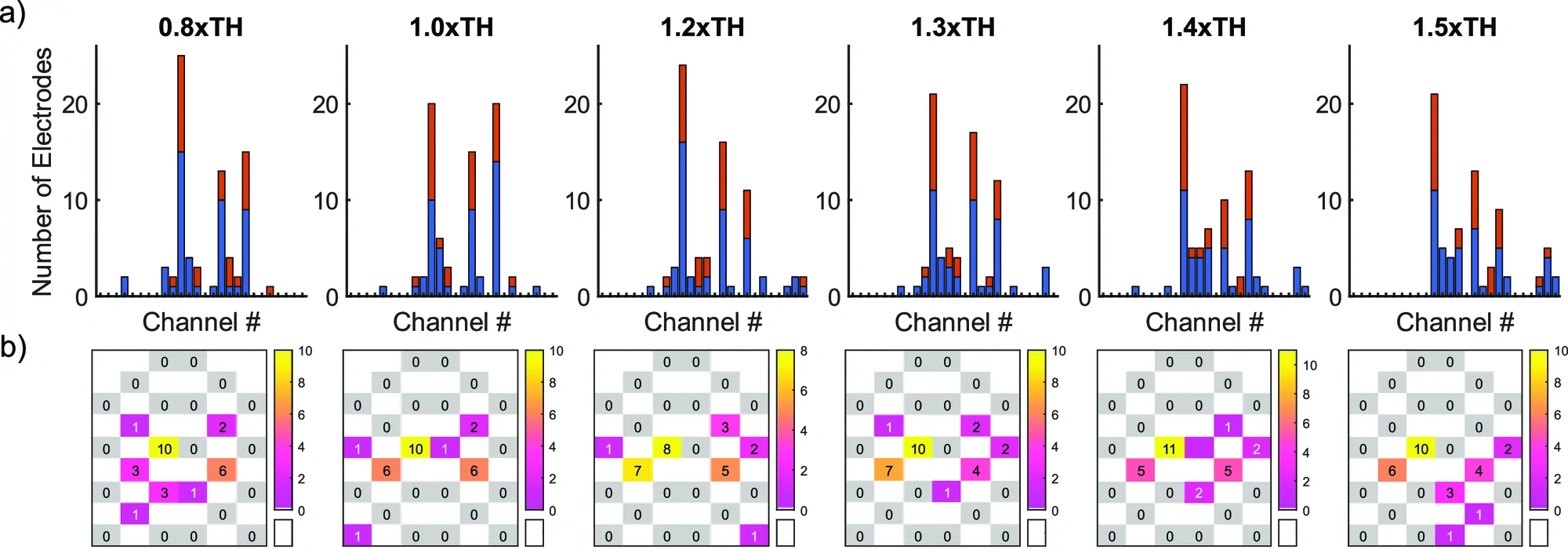
*Assessment of distribution of highest value EMG channel across all LIFEs for both stimulation configuration*. (a) Histograms showing the eEMG channel number with the highest value of the grid across different stimulation levels (xTH) for bipolar stimulation (red) and monopolar stimulation (blue). (b) Heatmaps from the histograms showing the position on the grid of the highest value distribution across all stimulation levels (only bipolar stimulation is shown). A concentrated heatmap indicates a consistent highest eEMG channel position, whereas a diffuse distribution reflects variability in the location of peak activation of the muscle across stimulation levels.

Figure 10 compares the frequency distributions of the channel exhibiting the highest RMS value with bipolar versus monopolar stimulation. Both stimulation types had similar distributions, with multiple peaks (figure 10(a)). However, compared with monopolar stimulation, the bipolar configuration showed a more concentrated distribution across fewer channels, as shown in 10(b), indicating that the channel with peak RMS values was more consistent than with monopolar stimulation.

## 4. Discussion

In this study, we examined how to obtain a more selective PNS by activating the intended nerve fiber sets while reducing off-target activation when using LIFEs. Two potential strategies were examined: electrode placement within the same fascicle and intrafascicular field steering. High-density epimysial EMG was used to quantify and map muscle activation patterns, providing a method to experimentally measure intrafascicular selectivity. Stimulation using LIFEs placed at different cross-sectional/longitudinal locations in the same fascicle can activate different muscle regions, indicating intrafascicular selectivity. Bipolar stimulation configurations can recruit fibers differently than monopolar configurations, offering a ‘lever’ to shape the activated sets of fibers within a fascicle. Both monopolar and bipolar stimulation evoked graded eEMG responses. Improving on-target selectivity is a path toward next-generation bioelectronic therapies with fewer side effects and better outcomes, potentially enabling more precise organ-specific neuromodulation.

### 4.1. HD-eEMG enables detailed assessment of intrafascicular selectivity

High-density epimysial EMG is a practical, minimally invasive method for assessing intrafascicular selectivity during PNS. Unlike traditional intramuscular EMG from multiple muscles that distinguishes between fascicles that innervate different muscles but lacks spatial mapping within a muscle, HD-eEMG enables detailed characterization of motor unit activity and spatial activation patterns within a muscle [32–36]. Our study shows that HD-eEMG combined with intrafascicular stimulation reveals spatially distinct and consistent muscle activation patterns, reflecting the selective recruitment of motor units based on electrode placement. This spatio-temporal information of M-wave responses to electrical stimulation allows the inference of activated sets of fiber subpopulations and demonstrates reliable, repeatable results across experiments.

### 4.2. Selective activation of nerve fiber subpopulations can be achieved by altering electrode placement

In this study, we evaluated intrafascicular selectivity when using LIFEs and demonstrated that different sets of nerve fibers within the same fascicle can be activated by altering electrode placement. Our results indicated that the muscle activation pattern varied depending on the electrode used for stimulation, despite all electrodes operating at comparable threshold levels (defined as the minimum current required to elicit a motor response). These variations in activation patterns can be attributed to the recruitment of different motor units (MUs) [32, 35, 37] that produce distinct motor response shapes across the HD-eEMG grid [36]. These HD-eEMG findings were consistent with the observed experimental behavior, where localized quasi-contractions were evident in specific muscle regions, and these regions shifted when stimulation was delivered through a different electrode. These differences in eEMG waveform morphology are influenced by multiple factors, including the number of active MUs, dispersion of innervation zones, MU location, and MU distribution [36, 38]. These variations were observed across all HD-eEMG channels, and AUC values were calculated from the profile curves. This distinction between muscle activation patterns elicited by intrafascicular electrodes was further supported by quantitative comparison across electrode pairs within the same fascicle (section 3.2.2), where pairwise spatial correlation between activation patterns was predominantly low around threshold, consistent with different LIFEs recruiting distinct sets of muscle fibers.

Conventional PNS approaches predominantly use cuff electrodes that wrap the targeted nerve and deliver an electrical charge across the entire nerve trunk [4, 13, 19, 39]. Although cuff electrodes are less invasive and exhibit long-term stability [9, 19, 39, 40], their limited spatial selectivity is a critical disadvantage. Cuff-based stimulation approaches often recruit multiple fiber subpopulations by stimulating a broad cross-section of the nerve, leading to unintended side effects [41]. For example, PNS using cuff electrodes to restore sensory feedback in users of upper-limb prosthetics has been associated with unintended muscle activation [42], whereas using cuffs for VNS can inadvertently activate the laryngeal muscles and reduce therapeutic tolerability [4, 43, 44].

In contrast, the geometric properties of LIFEs enable placement at various cross-sectional and longitudinal positions within a fascicle, thereby reducing the distance between the active site of the electrode and targeted nerve fibers [11, 17]. Fiber recruitment is governed by biophysical characteristics, where large myelinated fibers exhibit lower activation thresholds than smaller, unmyelinated fibers, making electrode placement critical. LIFEs can be positioned in proximity to sensory, motor, or autonomic fibers, each characterized by distinct thresholds, conduction velocities, and functional roles (i.e. thermoreceptors, somatic motor neurons, visceral afferents, and nociceptors) [45, 46]. These spatial relationships enable intrafascicular stimulation to achieve higher selectivity than extraneural approaches, although the potential risk of activating non-targeted fibers owing to imprecise placement is heightened.

Furthermore, when the stimulation amplitude was increased in our experiments, motor responses exhibited a graded increase, reflecting the recruitment of additional subpopulations as the current spread within the fascicle. These findings are consistent with the well-known principles of nerve stimulation: increasing the stimulation amplitude, and thereby the total charge delivered, enables recruitment of nerve fibers with higher thresholds and expands the recruitment of fibers positioned farther from the electrode.

Although stimulation amplitude significantly influenced AUC on average, the direction and pattern of its relationship with AUC differed substantially among LIFEs (section 3.2.2). These findings indicate the flexibility of intrafascicular stimulation using LIFEs and the wide range of activation patterns elicited by different electrode locations. This effect of stimulation amplitude was also evident when comparing activation patterns elicited by different LIFEs implanted within the same fascicle, where the proportion of highly correlated pairs increased at higher stimulation amplitudes, indicating that current spread increases overlap between the fiber populations recruited by neighboring electrodes. Future research to identify anatomical or other factors that influence the effects of LIFE location and stimulation amplitude may enable reliable and predictable control of activation patterns.

During this study, stimulation pulse trains were delivered to the gastrocnemius lateralis at tetanic frequencies low enough to produce a clear and reproducible muscle response while remaining below the threshold required to elicit maximal sustained contraction. This paradigm enabled accurate assessment of muscle patterns and minimized fatigue by preventing complete contraction-relaxation cycles, thereby allowing consistent evaluation of M-wave responses. Although intrafascicular selectivity was assessed using muscle activity (HD-eEMG), these findings may be translatable to sensory fiber activation. Prior research has demonstrated that intraneural electrodes for the PNS can evoke precise and proportional sensory feedback in humans. For example, individuals with upper-limb amputations who have received intraneural implants have reported restoration of distinct tactile percepts and an enhanced ability to discriminate objects [7, 18, 42, 47, 48]. The underlying biophysical principle remains unchanged: intrafascicular electrodes decrease the distance to the targeted axons, resulting in lower activation thresholds and limiting the spread of current. Therefore, the selectivity observed in motor activation is likely to be observed in sensory fibers. Hence, reducing the side effects of PNS-based therapies remains a significant challenge in the field of bioelectronic medicine. Enhancing the selectivity of the PNS paradigms is a promising approach for mitigating these undesirable effects.

Enhanced selectivity has the potential to improve the precision of the existing neurostimulation technologies. For instance, these results could inform PNS-based approaches for restoring sensory feedback in individuals with amputations by reducing off-target effects and improving and refining sensory inputs. Furthermore, improving selectivity may broaden the clinical and technological scope of PNS-based approaches, enabling the exploration of applications constrained by limited selectivity.

### 4.3. Electric field steering can be used for activation of additional fiber subpopulations

In our experiments, bipolar stimulation produced higher RMS values for motor responses than monopolar stimulation, indicating a stronger and more synchronized fiber activation. Although previous studies have suggested that bipolar stimulation decreases activation as electrode spacing increases [49], the spacing between multiple LIFEs in the sciatic nerve may have been insufficient to elicit this effect. Instead, these electrodes may act synergistically to generate a more concentrated electric field, resulting in enhanced activation. The use of LIFEs in a bipolar configuration revealed principles comparable to those observed for extraneural electrodes. Multiple LIFEs implanted within the same fascicle used in a bipolar configuration provided access to sets of fiber subpopulations that neither electrode could independently reach. However, while bipolar stimulation altered muscle activation patterns compared to monopolar stimulation, it did not significantly change the overall selectivity of recruitment as quantified by AUC. Instead, bipolar stimulation primarily redistributed the pattern of recruited muscle activity, suggesting that the electric field can be steered towards different nerve fiber sets.

Electric field steering is an additional strategy for selective activation of nerve fiber subpopulations. By employing multiple stimulation sites and adjusting the polarity and amplitude, the spatial distribution of the current within the nerve can be directed toward specific fiber groups [22, 24, 50]. Previous studies have used multi-contact cuff electrodes to enhance selectivity by shaping the electric field to target or avoid specific nerve regions. For example, Dali *et al* [23] evaluated various multi-contact cuff configurations, including tripolar, longitudinal, transverse, and steering current rings, and demonstrated that altering these could steer the current to activate specific muscles, with each configuration exhibiting distinct effects on selectivity, efficiency, and charge propagation. Computational modeling studies have similarly suggested that electric field steering could optimize VNS to modulate heart rate while reducing side effects compared to standard ring cuff configurations [51].

Our bipolar configuration findings demonstrate that implanting multiple LIFEs within the same fascicle could enable additional stimulation strategies that cannot be achieved with a single electrode. Thus, bipolar stimulation could provide an additional degree of flexibility for targeting different fiber sets than those using monopolar stimulation alone. By alternating and evaluating different electrode combinations, it is possible to refine the stimulation paradigms tailored to specific recruitment patterns. Ultimately, this approach may enhance selectivity by steering activation across multiple electrodes, thus providing a more precise and adaptable method for targeting distinct fiber subpopulations.

## 5. Limitations

Although LIFEs have been successfully implanted in humans for clinical applications, such as restoration of sensory feedback in individuals with amputations, there are currently no surgical techniques that enable precise implantation of intraneural electrodes to target a pre-specified fiber population. Currently, this limitation extends to general applications but was also present in our studies. Implantation of intrafascicular electrodes typically relies on morphological data from prior studies [52, 53] without real-time confirmation of electrode positioning. As demonstrated in this study, electrode placement is critical for achieving intrafascicular selectivity; even minor positional differences can substantially alter the subset of activated fibers. Therefore, the development of surgical techniques or guidance systems that allow high-precision electrode placement can significantly reduce the off-target effects.

In our study, each electrode implanted within a fascicle occupied a different longitudinal and transverse intrafascicular location. Although the electrodes’ entry points were visually identified as separate, their specific intrafascicular location was not confirmed with histology. Performing histology of nerve tissue with multiple wires within a fascicle is technically challenging. Nevertheless, future studies could use histological assessment of electrode separation to examine how intrafascicular location relates to HD-eEMG spatial activation patterns and how electrode-to-electrode distance influences responses to bipolar stimulation.

Another limitation is that we did not directly measure activation of nerve fibers. Our method for assessing intrafascicular recruitment from HD-eEMG activation patterns is indirect, so we can make only inferences about selectivity given that distinct HD-eEMG patterns are consistent with selective recruitment of different motor fibers. Direct axon-level confirmation of recruitment selectivity, for example using refractory interaction [54, 55], was not performed in this study. Refractory interaction requires precisely timed paired-pulse stimulation between electrodes that was not part of our stimulation protocol (section 2.5). Incorporating this methodology or a comparable direct electrophysiological method, such as electroneurograms, in future studies could provide more granular information to understand how nerve fiber recruitment is affected by stimulation conditions.

LIFEs, like other penetrating intraneural electrodes, are subject to chronic tissue encapsulation, micromotion relative to surrounding tissue, and mechanical fatigue at the electrode-tissue interface, all of which could influence stimulation performance over time [11, 19, 56]. Electrodes made using thin-film technologies can also face delamination [57]. Cumulatively, these factors have limited the therapeutic use of intraneural electrodes to periods shorter than would be required for chronic neuromodulation applications. Hence, translating these stimulation strategies to enhance selectivity with LIFEs will require continued assessment over long-term studies for safety and feasibility of intrafascicular electrode use, for example in Clinical Trial # NCT03432325, and perhaps development of novel electrode materials and encapsulation-resistant coatings [58, 59].

Furthermore, although LIFEs have been used in clinical trials, they have not yet received FDA approval as a therapeutic neural interface. Consequently, there are a limited number of research groups working to improve implantation techniques or optimize stimulation paradigms that use LIFEs.

## 6. Conclusions

Enhancing the selectivity of PNS to minimize undesirable side effects continues to pose a significant challenge to bioelectronic medicine. Using multiple LIFEs implanted within the sciatic nerve, we investigated the effects of intrafascicular electrode placement and field steering on stimulation selectivity. We demonstrate that HD-eEMG is a robust method for evaluating intrafascicular selectivity. Our results indicate that both approaches can selectively activate discrete subpopulations of nerve fibers within a fascicle, improving stimulation specificity and potentially reducing off-target activation in bioelectronic medical therapies.

## Data Availability

The data that support the findings of this study are openly available at the following URL/DOI: https://doi.org/10.5061/dryad.rbnzs7hth.
